# MCP’s New Editorial Posture Moving Forward

**DOI:** 10.1016/j.mcpro.2023.100646

**Published:** 2023-09-27

**Authors:** Al Burlingame

I am delighted to recognize the appointment of Anne-Claude Gingras as the new Director of the Lunenfeld-Tanenbaum Research Institute in Toronto. In addition to her long-standing role as Deputy Editor of our journal, for which we extend our heartfelt thanks, she has developed her impressive career at Lunenfeld since 2005, earning significant national and international recognition. For example, she is listed among the world's top female scientists and received an Associate Membership in the European Molecular Biology Organization. Her notable international awards include the HUPO Discovery in Proteomics Science Award and the MCP Lectureship Award. At the national level, she has been honored with the Canadian National Proteomics Network-Tony Pawson Proteomics Award and the Charles W. Gowdey Distinguished Lecture Award, among others. She is a fellow of the Royal Society of Canada. We will sorely miss her many insights and contributions to the editorial leadership of MCP.

I would also like to take this opportunity to inform our readership of several additional editorial changes taking place that will enable us to focus on serving the growing scientific community in China and Asia and on strengthening our long-standing contributors from Europe. I am delighted to recognize the appointments of Lan Huang and Tiannan Guo as additional Associate Editors of MCP. Lan Huang is a world-class scientist in mass spectrometry–based proteomics at the University of California, Irvine. She will strengthen our commitment to the growing field of mass spectrometric–based proteomic methodologies and research into the structural elucidation of protein complexes and machines, even in whole cells. In addition, Lan has been involved in the leadership of the Chinese–American Mass Spectrometry Society ((2023) **Molecular & Cellular Proteomics** *22* (6), 100559) and will be a leader in our expansion in mainland China together with our other new Associate Editor, Tiannan Guo. Tiannan Guo studied clinical medicine at Tongi Medical College and received his MD in 2006. He then studied biology and cancer proteomics in Singapore, receiving his PhD in 2012. Tiannan carried out postdoctoral work with Ruedi Abersold at Eidgenössische Technische Hochschule Zürich and eventually joined the Westlake Institute for Advanced Studies as a tenure-track faculty member in 2017.

Finally, I am also pleased to announce the appointment of Bernhard Küster as Deputy Editor effective immediately. Until now, Bernhard has been serving as an outstanding Associate Editor of MCP. He is a major academic leader at Technische Universität München and an accomplished contributor and talent at the forefront of mass spectrometry–based proteomics in Europe. He is well known throughout the research community and is recognized for his seminal works on, for example, kinase drug crossreactivity and regulation by post-translational modifications, among many additional contributions ((2023) **Science** *380*, 93–101; (2017) **Science** *358*, eaan4368). In a recent technical feat, his team developed Prosit, a proteome-wide prediction tool of peptide tandem mass spectra by deep learning ((2019) **Nat. Methods** *16*, 509–518). This year he received the “Distinguished Achievement in Proteomic Sciences Award,” from the Human Proteome Organization. He has also co-chaired the last two highly successful “Mass Spectrometry in the Health and Life Sciences” symposia, integrations of biological challenges and cutting-edge technologies, with Steve Carr and myself in San Francisco and Cambridge in 2022.

Please join me in welcoming these changes that will position MCP to attract increasing global submissions of the field’s best research to MCP.

**Figure:**
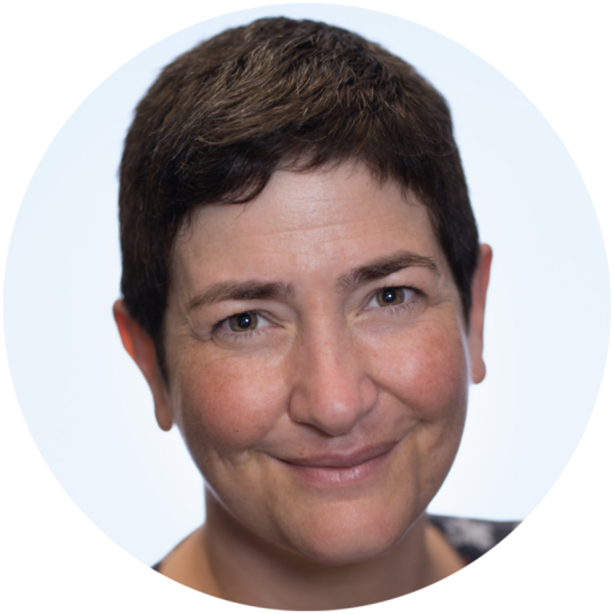
Anne-Claude Gingras

**Figure:**
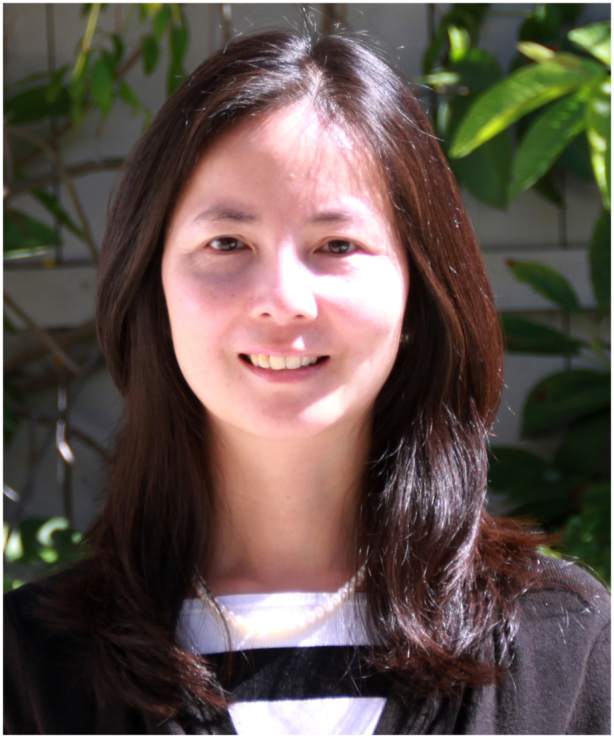
Lan Huang

**Figure:**
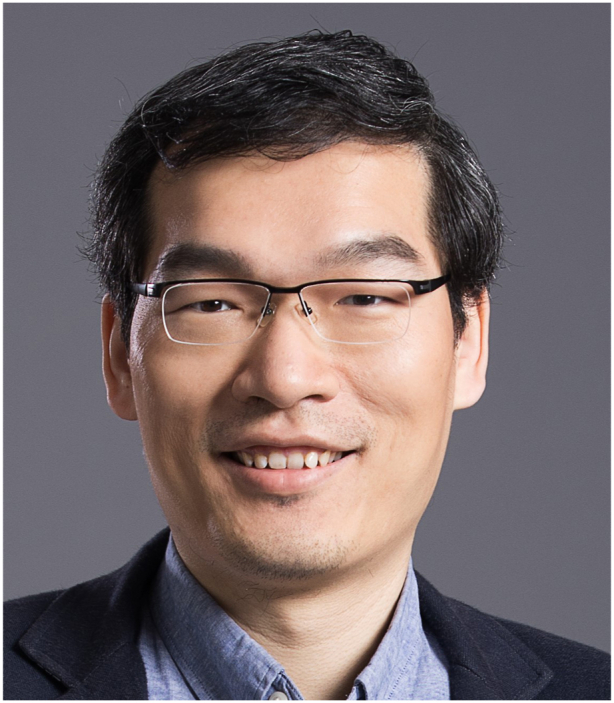
Tiannan Guo

**Figure:**
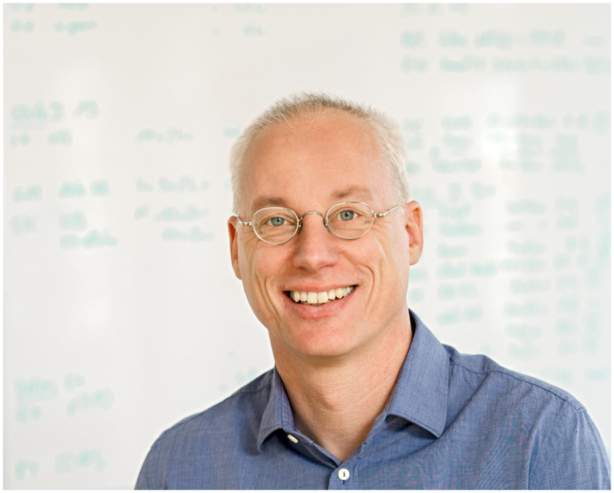
Bernhard Küster

